# Clinical Aspects of Foot Health in Individuals with Alzheimer’s Disease

**DOI:** 10.3390/ijerph15020286

**Published:** 2018-02-07

**Authors:** Daniel López-López, Marta Grela-Fariña, Marta Elena Losa-Iglesias, César Calvo-Lobo, David Rodríguez-Sanz, Patricia Palomo-López, Ricardo Becerro-de-Bengoa-Vallejo

**Affiliations:** 1 Research, Health and Podiatry Unit, Department of Health Sciences, Faculty of Nursing and Podiatry, Universidade da Coruña, 15403 Ferrol, Spain; daniellopez@udc.es (D.L.-L.); martagrela95@gmail.com (M.G.-F.); 2 Faculty of Health Sciences, Universidad Rey Juan, 28922 Carlos, Spain; marta.losa@urjc.es; 3 Nursing and Physical Therapy Department, Institute of Biomedicine (IBIOMED), Universidad de León, 24401 Ponferrada, Spain; 4 Research Group, Faculty of Health, Exercise and Sport, European University of Madrid, 28670 Villaviciosa de Odón, Spain; davidrodriguezsanz@gmail.com; 5 University Center of Plasencia, Universidad de Extremadura, 06071 Badajoz, Spain; patibiom@unex.es; 6 School of Nursing, Physiotherapy and Podiatry, Universidad Complutense de Madrid, 28040 Madrid, Spain; ribebeva@ucm.es

**Keywords:** Alzheimer’s disease, foot diseases, joint flexibility, musculoskeletal system, shoes

## Abstract

Alzheimer’s disease (AD) shows a marked presence of physiologic changes and the start or aggravation of underlying diseases such as physical frailty in diverse anatomical regions. It is believed to have a particularly harmful effect on the health of the foot. We examined the foot health status in older persons with AD, with a specific focus on the extent to which people with AD may be using inadequate footwear in old age. Seventy-three community-dwelling people with probable, mild to moderate AD aged 65–95 years were recruited from a center of excellence for AD. A single trained physician evaluated health status and foot conditions. Current shoe and foot length and width measurements were taken using a calibrated Brannock device. The results indicate that sixty-five participants (89.04%) suffered from feet problems. Also, only twenty-two subjects (30.14%) used the correct shoes in width and size related with the morphology of their feet. Fifty-one participants (69.86%) were using incorrect shoes in length or width. The present study revealed that peoples with AD had a high presence of foot health problems. Also, the use of inappropriate shoes revealed measurable differences of association between shoe size and the morphology of the foot.

## 1. Introduction

Alzheimer’s disease (AD) is a progressive neurodegenerative disorder more common in the elderly and is a growing epidemic worldwide [1], affecting approximately 4.4% to 9.7% of elderly people and is predicted to double every 20 years until at least 2040 [2,3,4]. AD is associated with negative outcomes including short-term memory troubles [5], problems in walking [6], changes in equilibrium and limb coordination [7], falls [8], sleep disturbance [9], and physical frailty [10].

Although the clinical aspects of foot health in AD people are evident, the link with AD is unclear, but it is believed to be influenced by the functional, social, and cognitive ability of these people. Recent studies by López-López et al. in older people without AD have demonstrated the influence of decreased sensitivity and the absence of regular foot checks, which can contribute to use of inadequate footwear [11]. Also, López-López et al. confirmed that incorrect shoe size has a significant negative impact on quality of life related to foot health in elderly individuals [12].

Despite this, no studies have been carried out so far to analyze foot health status in older persons with AD, who may favor the use of inadequate footwear. This is an important factor to consider for the prevention of falls, postural alterations, foot disorders, and other basic illnesses in order to ensure a better quality of life and wellbeing for people with AD.

In order to verify this hypothesis, the aim of this study was to examine foot health status in older persons with AD, and more specifically, the extent to which persons with AD may be using inadequate footwear in old age.

## 2. Materials and Methods

### 2.1. Design and Sample

Seventy-three subjects diagnosed with AD living in a “center of excellence” for AD participated in the study between January 2017 and April 2017. It was a cross-sectional research project and a consecutive sampling method was used to select the participants. The inclusion criteria was: aged 65 or more and legal guardians having provided informed consent. The exclusion criteria included having a non-AD-related medical condition that might affect balance control, trauma or history of lesions of limbs (hip, knee, ankle, and foot), sleep disorders, being immunocompromised, refusal to of the legal guardian (s) to sign the consent form or being incapable of understanding the instructions necessary to carry out the present research.

### 2.2. Procedure

All initial measurements of the subjects were related to demographic characteristics (age and gender). Predisposing factors were determined from medical history records using an identical protocol and were carried out by the same trained physician prior to the assessment.

Secondly, activities of daily living in relation to personal care and mobility of the patient were evaluated using the Barthel Index (BI). This used a validated instrument and showed high mark reliability ratings, with 0.89 test-retest reliability and 0.95 internal reliability [13]. It was used to determine independent physical functioning and gives a score ranging from 0 to 100. A zero score represents the worst state of independent physical functioning and 100 is the best possible condition.

In the third place, anthropometric features were measured, including the height (cm), weight (kg) with the participant barefoot and wearing light clothing, and the body mass index (BMI) was calculated from the height (m) and weight (kg) by applying Quetelet’s equation [14]:

BMI = weight/height^2^

Finally, a validated Brannock Device-type^®^ (BD) (Algeos.com, Sheridan House, Bridge Industrial Estate, Liverpool, UK) measuring instrument was then used to measure foot length and width; this instrument has previously been shown to be reliable, with an intraclass correlation coefficient (ICC) of 0.96–0.99 [15]. Each patient stood in a relaxed posture, barefoot and with feet slightly apart and their weight evenly distributed. The researcher helped them to place their foot correctly in the device, with the heel located against the back of the heel cup, and measured the distance to the end of the longest toe (which is not necessarily the first toe) [15,16]. The identical protocol was used for the other foot and this data was used to measure shoe size.

The sample size was calculated with the software from Unidad de Epidemiología Clínica y Bioestadística. Complexo Hospitalario Universitario de A Coruña. Universidade da Coruña (www.fisterra.com) [17]. The calculations were based on the number of individuals with AD in the state of Galicia, which has a total population of 2,718,525 persons (https://www.ige.eu/web/index.jsp?paxina=001&idioma=gl), with 13.8% of the population having AD. The calculations used a 2-tailed test, an α level of 0.03, and a desired power analysis of 85% with a β level of 3% and a precision of ±3%. As such, assuming an information loss of 15%, at least 67 cases were determined to be required for the study. Ultimately, 73 people were included in the study.

### 2.3. Ethical Considerations

This research was approved by the Research and Ethics Committee of the University of A Coruña, file number CE 3/2017. All the parents and/or legal guardians gave their informed consent before the persons with AD concerned were included in the study. Ethical standards for research on human beings based on the Declaration of Helsinki (World Medical Association) and the Convention of the Council of Europe on human rights and biomedicine, as well as those based on the Universal Declaration of the United Nations Educational, Scientific and Cultural Organization on the Human Genome and Human Rights and other appropriate national or institutional organizations were preserved.

### 2.4. Statistical Analysis

Categorical variables are shown as absolute values and percentages, whereas the quantitative variables described are the mean, standard deviation (SD), and maximum and minimum values. The Fisher’s exact test was used to compare the categorical variables of foot and shoe measurements.

All variables were examined for normality of distribution using the Shapiro-Wilk test, and data were considered normally distributed if *p* > 0.05. Independent Student *t*-tests were performed to find if differences between males and females with AD were statistically significant when showing a normal distribution (age, weight, height, and BMI). Measurements which were not normally distributed (BI) were tested using the non-parametric U Mann-Whitney test to examine differences between male and female groups with AD.

In all of the analyses, statistical significance was established with a *p*-value < 0.05. All the analyses were performed with commercially available software (SPSS 19.0, IBM, Chicago, IL, USA).

## 3. Results

All variables showed a normal distribution (*p* > 0.05) except for BI (*p* < 0.05). A total of 73 participants completed all the stages of the research process, 25 of whom were men (34.25%) and 48 women (65.75%). Their ages ranged from 65 years to 95 years (mean = 81.40 ± 6.45 years). BMI ranged from 14.71 to 32.48 (mean = 24.84 ± 3.46) and BI mean 74.71 ± 21.52 (Table 1).

A majority of participants (89.04%; *n* = 65) stated that they suffered from foot problems and a subsequent physical examination revealed that 79.45% (*n* = 58) had bunions, 52.05% (*n* = 38) had nail or keratotic disorders, 47.9% (*n* = 35) had hallux limitus, 19.2% (*n* = 14) had hallux rigidus, and 12.3% (*n* = 9) had deformed toes. After measuring foot and shoe size, we determined that 69.86% of participants were using differing shoe sizes between the left and right and inadequate footwear. As shown in Table 2, only twenty-two subjects (30.14%) used shoes that met the needs and requirements of their feet. Further, although twenty-two individuals (30.14%) were wearing shoes that were of the correct length, they were of insufficient width.

## 4. Discussion

We examined foot health status in older persons with AD, and the extent to which they may be using inadequate footwear in old age.

As AD progresses the sense of balance is likely to deteriorate, with an associated risk of falling, and self-care will become increasingly difficult. Thus, the control foot is considered by physicians and podiatrists to be very important for optimizing vigilance for early signs of foot injury and inadequate hygiene largely due to the high prevalence of the at-risk foot. Indeed, foot health has been recognized as a potential threat to public health, as stated by Najafi et al., who described the age-related decline in foot strength and flexibility and the emerging evidence that foot problems increase the risk of falls, established guidelines for falls prevention, and recommended that older adults have their feet examined by a podiatrist as a precautionary measure [18].

Our results revealed that people with AD had a high presence of feet problems, with similar results to those of the few studies carried out on this matter in older people without AD. For example, Chaiwanichsiri et al. showed that 87% of older persons presented with foot deformities that increased the risk of falls even in healthy older persons [19].

López-López et al. also declared that inadequate footwear is a very common cause of injury in older people and people with rheumatoid arthritis, and that this factor increases the likelihood of shoes causing grievous bodily harm [11,16]. These results are similar to our research, which indicates that only twenty-two subjects (30.14%) used correct shoes in width and size related to the morphology of their feet. Fifty-one participants (69.86%) were using incorrect shoe lengths or widths, thus highlighting the need for regular foot care and monitoring in people with AD.

These results further highlight what may appear to be obvious: that assessing the feet of people with AD must also include an assessment of the footwear that they wear. Indeed, future clinical trials with novel interventions like education programs for families of individuals with AD or the use of the Brannock Device-type measuring instrument may improve the use of adequate shoes according to the foot lengths and widths of subjects with AD.

Nevertheless, there are several limitations to the study that should be acknowledged. A larger and more diverse (individuals from various countries) sample size would be beneficial to improve the strength of the study and identify other factors involved. Despite having provided a sample size calculation, a power analysis based on the results of a prior pilot study would be beneficial to improve the research quality. Also, there was only one single evaluator analyzing the participants’ feet. Lastly, future studies should have at least two blinded evaluators. One evaluator for feet examination and another for footwear convenience in order to compare the blinded data that is recorded. Also, data comparison with a blinded control group without AD would have strengthened the results. This study provides limited statistical analysis, for example, looking at only gender as an independent variable and lack of information regarding the subjects’ demographics, characteristics, and foot problems, which should be considered in future studies.

Most importantly, further continuous research is required in this area in order to find out about the different therapeutic interventions that could be used by professionals of podiatry and general medicine in order to potentially improve both the general health and foot health in people with AD.

## 5. Conclusions

The present study revealed that people with AD had a high presence of foot problems. Also, they often used inappropriate shoes, with measurable differences noted between shoe size and the morphology of the foot.

## Figures and Tables

**Table 1:** Socio-demographic and clinical characteristics of the sample with AD.

Demographic Characteristics	Total Group Mean ± SD Range *n* = 73	Male with AD Mean (SD) Range *n* = 25	Female with AD Mean (SD) Range *n* = 48	*p*-Value
Age, years	81.40 ± 6.45 (65–95)	78.52 ± 6.04 (65–87)	82.90 ± 6.2 (69–95)	0.010 *
Weight (kg)	66.22 ± 12.21 (36.2–97.2)	74.60 ± 11.15 (43–97.2)	61.85 ± 10.4 (36.2–86)	0.001 *
Height (cm)	162.93 ± 9.84 (136–189)	170.12 ± 9.01 (155–189)	159.19 ± 8.07 (136–175)	0.001 *
BMI (kg/m^2^)	24.84 ± 3.46 (14.71–32.48)	25.82 ± 3.58 (14.71–32.48)	24.34 ± 3.33 (18.29–32.42)	0.001 *
BI	74.71 ± 21.52 (0–100)	78.76 ± 24.21 (0–100)	72.60 ± 19.92 (10–100)	0.001 **

Abbreviations: AD, Alzheimer disease, BMI, body mass index; SD, standard deviation; BI, Barthel Index. In all the analyses, *p* < 0.05 (with a 95% confidence interval) was considered statistically significant. * Student t test for independent samples was used for the analysis data. ** Mann-Whitney U test was utilized for the analysis data.

**Table 2:** Foot and shoe measurements (both feet, standing position).

Standing Position	Excessive Shoe Width	Correct Shoe Width	Insufficient Shoe Width	Total	*p*-Value
Right Foot					
Shoe size too big	0	6	22	28	0.003 *
Correct shoe size	3	22	20	45	
Shoe size too small	0	0	0	0	
Total	3	28	42	73	
Left Foot					
Shoe size too big	0	7	20	27	0.0013 *
Correct shoe size	1	22	22	45	
Shoe size too small	0	1	0	0	
Total	1	30	42	73	

In all the analyses, *p* < 0.05 was considered statistically significant. * Fisher’s exact test was used.
